# A protocol to visualize on-target specific drug binding in mammalian tissue with cellular resolution using tissue clearing and click chemistry

**DOI:** 10.1016/j.xpro.2022.101778

**Published:** 2022-10-25

**Authors:** Zhengyuan Pang, Li Ye

**Affiliations:** 1 Department of Neuroscience, The Scripps Research Institute, La Jolla, CA 92037, USA; 2 Department of Molecular Medicine, The Scripps Research Institute, La Jolla, CA 92037, USA

**Keywords:** Neuroscience, Biotechnology and bioengineering

## Abstract

Here, we provide a protocol to visualize on-target specific drug binding in mammalian tissue with cellular resolution. By combining tissue clearing and click chemistry, this protocol allows fluorescence tagging of covalent drug binding *in situ*. In addition, the protocol is compatible with molecular marker staining for cell type identifications.

For complete details on the use and execution of this protocol, please refer to Pang et al. (2022).

## Highlights

• A protocol to visualize PF7845-yne binding in mammalian brain sections
• Whole-body perfusion to remove blood
• Lipid removal by tissue clearing to render tissue transparent
• Drug *in situ* fluorescence labeling with click chemistry

## Abstract (editor highlights)

Publisher’s note: Undertaking any experimental protocol requires adherence to local institutional guidelines for laboratory safety and ethics.

## Graphical abstract

**Figure:**
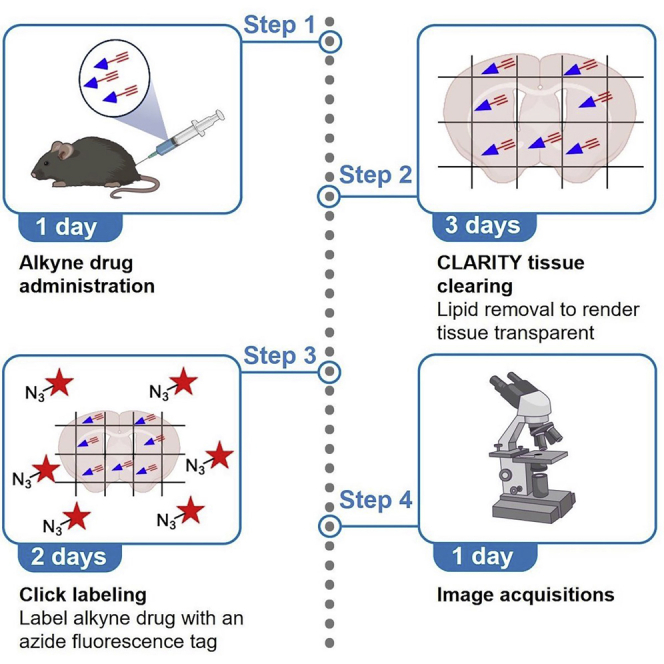

## Before you begin

Understanding drug actions in vivo is critical for developing effective therapies. Despite remarkable methodological advancement has been made to profile the drug-target interactions at the molecular level, a detailed understanding at the cellular level has not been established. Conventional strategies studying drug tissue distribution typically involves homogenizing the tissue/organ of interests, during which the spatial or cellular information is lost. Position emission tomography (PET) is widely utilized to study spatial drug distribution but lacks the resolution to resolve drug binding at cellular level. Given the high degree of cell type heterogeneity of mammalian tissue, especially in the central nervous system (CNS), it is desirable to visualize drug binding with cellular resolution, while maintaining compatibility with molecular characterizations.

To profile drug binding targets, drugs can be delicately modified with an alkyne handle. With copper(I)-catalyzed azide alkyne cycloaddition (CuAAC) click reaction, a tag (such as biotin or fluorophore) can be introduced for proteomic scale analysis. Such a strategy has proven highly versatile in chemoproteomics studies (Parker and Pratt, 2020). However, direct click labeling in mammalian tissue for in situ drug mapping has been challenging due to potential side reaction and low signal to noise ratio (SNR). Herein, by integrating tissue clearing and click chemistry drug labeling, we addressed these challenges with CATCH, a newly developed strategy to visualize on target specific covalent drug binding with high resolution.

In this protocol, we describe the specific steps for visualizing PF7845-yne binding in 100 μm mouse brain tissue sections. PF7845 is a highly selective fatty acid amide hydrolase (FAAH) inhibitor (Johnson et al., 2011). Its alkyne analog PF7845-yne has well-characterized in previous chemoproteomics studies (Niphakis et al., 2012). In addition to PF7845, we have successfully mapped another FAAH inhibitor BIA10-2474 (Huang et al., 2019), a monoacylglycerol lipase (MAGL) inhibitor MJN110 (Chang et al., 2013), and a monoamine oxidase (MAO) inhibitor Pargyline (Krysiak et al., 2012). The synthesis and characterization of these probes, as well as any new probes the users would like to use, should be separately carried out with chemoproteomics studies. The protocol here only focuses on the histological and imaging applications of existing, pre-validated probes.

### Institutional permissions

All experimental protocols were approved by the Scripps Research Institute Institutional Animal Care and Use Committee and were in accordance with the guidelines from the National Institute of Health.

### Preparation of tilted tube rack

**Timing: 10 min**

**Figure 1:**
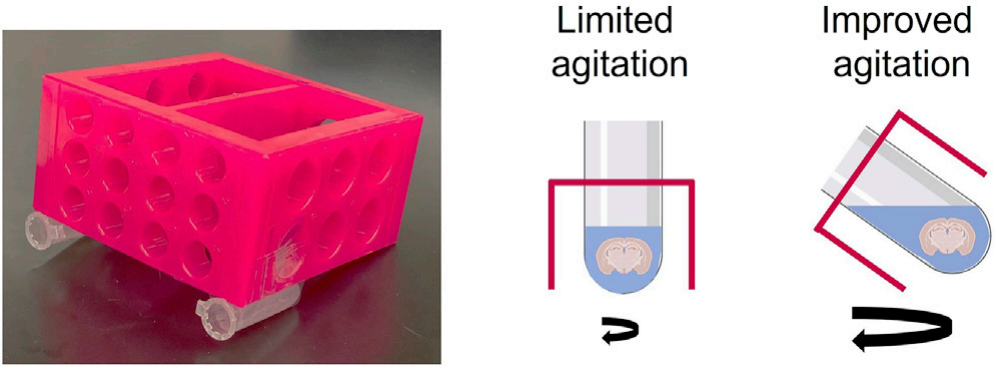
Custom made tilted rack for click reaction When tubes are placed on the rack, it allows maximal agitation in a small buffer volume.

1. Use two 2 mL Eppendorf tubes, remove the cap.
2. Attach the tubes to an autoclavable 4-way test tube rack with tapes (Figure 1). This rack is specifically made for click reaction incubation and reaction. As the rack is tilted, it allows maximal agitation in a small reaction volume.

## Key resources table

**Table:** 

REAGENT or RESOURCE	SOURCE	IDENTIFIER
**Antibodies**		

anti-FAAH (1:400 dilution)	Abcam	Cat#ab54615; RRID: AB_2101890
Alexa Fluor 488 F(ab’)2 Fragment Donkey anti-Mouse IgG (1:600 dilution)	Jackson Immuno Research	Cat#715-546-150; RRID: AB_2340849

**Chemicals, peptides, and recombinant proteins**		

Alexa-647 picolyl azide	Click Chemistry Tools	Cat#1300-5
PF7845-yne	Ye lab (Niphakis et al., 2012)	N/A
3-[4-({bis[(1-tert-butyl-1H-1,2,3-triazol-4-yl)methyl]amino} methyl)-1H-1,2,3-triazol-1-yl]propanol (BTTP)	Click Chemistry Tools	Cat#1414-100; CAS#1334179-85-9
Dimethyl sulfoxide (DMSO)	Sigma-Aldrich	Cat#D8418; CAS#67-68-5
Copper sulfate	Sigma-Aldrich	Cat#C1297; CAS#7758-98-7
Sodium ascorbate	Sigma-Aldrich	Cat#A4034; CAS#134-03-2
Isoflurane	Covetrus	Cat#11695067772
Agarose	Sigma-Aldrich	Cat#A6013-500G; CAS#9012-36-6
20× saline-sodium citrate (SSC) buffer	VWR	Cat#10128-690
Tween-20	Sigma-Aldrich	Cat#P2287-500ML; CAS#9005-64-5
Tween-80	Sigma-Aldrich	Cat#P4780-100ML; CAS#9005-65-6
Saline (0.9 M sodium chloride solution)	Sigma-Aldrich	Cat#S8776; CAS#7647-14-5
EMS perfusion fixative reagent (4% paraformaldehyde, PFA)	Fisher Scientific	Cat#5033441
Hybridization Chain Reaction (HCR) probe hybridization buffer	Molecular Instruments	N/A
HCR probe washing buffer	Molecular Instruments	N/A
HCR amplification buffer	Molecular Instruments	N/A
4′,6-diamidino-2-phenylindole (DAPI)	Sigma-Aldrich	Cat#D9542; CAS#28718-90-3
40% acrylamide solution	Bio-Rad	Cat#1610140
2% bis-acrylamide solution	Bio-Rad	Cat#1610142
32% PFA solution	Electron Microscopy Sciences	Cat#15714-S
Phosphate buffer saline (PBS), 10×, pH 7.4	Fisher Scientific	Cat#70-011-044
PBS, 1×, pH 7.4	Fisher Scientific	Cat#10-010-023
20% sodium dodecyl sulfate (SDS) solution	Fisher Scientific	Cat#50-488-742; CAS#151-21-3
VA-044 initiator	Fisher Scientific	Cat#NC0632395; CAS#27776-21-2
RapiClear, RI 1.45	SunJin Lab	Cat#RCCS005
TritonX-100	Sigma-Aldrich	Cat#X100-500ML; CAS#9036-19-5
Sodium azide	Sigma-Aldrich	Cat#S2002-100G; CAS#26628-22-8
0.5 M Ethylenediaminetetraacetic acid (ETDA), pH 8.0	Thermo Fisher Scientific	Cat#AM9260G

**Critical commercial assays**		

HCR signal amplification kit	Molecular Instruments	https://store.molecularinstruments.com/new-bundle/rna-fish

**Experimental models: Organisms/strains**		

Mouse: C57BL6J (5–8 weeks old, either sex)	The Jackson Laboratory	#000664

**Oligonucleotides**		

Somatostatin (SST) B1 HCR probe	Molecular Instruments	N/A
Alexa Fluor 488 HCR B1 hairpin	Molecular Instruments	N/A

**Software and algorithms**		

Fiji-ImageJ	(Schindelin et al., 2012)	https://imagej.net/software/fiji/

**Other**		

BUCHI Vac V-500 vacuum pump	Marshall Scientific	https://www.marshallscientific.com/Buchi-V-500-Vacuum-Pump-p/bu-v500.htm
SP BEL-ART polycarbonate vacuum desiccator	SP Bel-Art	Cat#F42025-0000
Innova 2000 shaker	Thomas Scientific	Cat#14278105
222DS benchtop shaking incubator	Thomas Scientific	Cat#1186N33
Olympus FLUOVIEW FV3000 confocal microscope	Olympus	https://www.olympus-lifescience.com/en/laser-scanning/fv3000/
Ismatec® Reglo Peristaltic Pump (perfusion pump)	Ismatec	http://www.ismatec.com/int_e/pumps/t_reglo/reglo.htm
Pump tubing, 3-stop, 2.79 mm ID	Masterflex	Cat#HV-96464-48
XLUMPlanFI, Olympus 10×, 0.6 NA water immersion objective	Olympus	N/A
Leica VT1000S Vibratome	Leica	https://www.leicabiosystems.com/us/research/vibratomes/leica-vt1000-s/
Autoclavable 4-way test tube rack	Cole-Parmer	Cat#EW-06733-00
2 mL Eppendorf centrifuge tube	Fisher Scientific	Cat#022-36-335-2
Screw top 5 mL Eppendorf tube	Eppendorf	Cat#0030122305
15 mL centrifuge tube	VWR	Cat#89039-664
50 mL centrifuge tube	VWR	Cat#89039-656
Parafilm	Fisher Scientific	Cat#13-374-10
Fisher Adhesive Microscope Slide	Fisher Scientific	Cat#12-550-15
Microscope Slide Cover Glass #1, 22 × 40 mm	Electron Microscopy Sciences	Cat#72200-31
Microscope Slide Cover Glass #1, 22 × 30 mm	Electron Microscopy Sciences	Cat#72200-21
Sally Hansen Xtreme Wear Nail Polish, Invisible, 0.4 Fl. Oz.	Amazon	N/A
Tough cut surgical scissors	F.S.T.	Cat#14130-17
Forceps	F.S.T.	Cat#11051-10
Suture scissors	ST Dental	Cat#C-0622
Insulin syringe & needle, ½ cc, 29G × ½ in.	Fisher Scientific	Cat#14-841-32
Needle (26G × ½)	Fisher Scientific	Cat#14-826-15
Kimwipe	Fisher Scientific	Cat#34120

## Materials and equipment

**Table:** PF7845-yne stock solution

Reagent	Final concentration	Amount
PF7845-yne	5 mg/mL	5 mg
DMSO	N/A	1 mL
**Total**	**N/A**	**1 mL**

Aliquot PF7845-yne stock solution. Aliquots can be stored in −20°C for up to a year. Avoid repeated freezing and thawing.

**Table:** Tween-80 stock solution

Reagent	Final concentration	Amount
Tween-80	25%	10 mL
dH_2_O	N/A	30 mL
**Total**	**N/A**	**40 mL**

Tween-80 stock solution can be stored in RT for up to a year.

**Table:** Drug administration solution

Reagent	Final concentration	Amount
PF7845-yne (5 mg/mL in DMSO)	0.1 mg/mL	4 μL
DMSO	10%	16 μL
25% Tween-80	2%	16 μL
Saline	N/A	164
**Total**	**N/A**	**200 μL**

Drug administration solution should be prepared fresh each time.

**Table:** A1P4 CLARITY solution

Reagent	Final concentration	Amount
40% acrylamide solution	1%	5 mL
2% bis-acrylamide solution	0.0125%	1.25 mL
VA-044 initiator	0.25%	0.5 g in 5 mL dH_2_O
32% paraformaldehyde	4%	25 mL
10× PBS	1×	20 mL
dH_2_O	N/A	∼144 mL
**Total**	**N/A**	**200 mL**

Components for A1P4 solution are kept in 4°C. A1P4 solution should be prepared fresh on ice prior to use and can be stable in 4°C for up to a week.

**CRITICAL:** Components for A1P4 CLARITY solution should be cooled to 4°C prior to use. Paraformaldehyde, acrylamide and bis-acrylamide are toxic. Avoid direct skin contact. Prepare A1P4 CLARITY solution in a chemical fume hood.

**Table:** PBS-SDS clearing solution

Reagent	Final concentration	Amount
20% SDS	8%	400 mL
10× PBS	1×	100 mL
dH_2_O	N/A	500 mL
**Total**	**N/A**	**1,000 mL**

20% SDS can be purchased as a stock solution. Please follow manufacturer instruction for storage conditions. Homemade 20% SDS stock solution should be checked for potential precipitation before use. Once made, 8% SDS clearing solution can be stored in RT for at least a year.

**Table:** PBS-NaN_3_ storage buffer

Reagent	Final concentration	Amount
NaN_3_	0.02%	0.2 g
10× PBS	1×	100 mL
dH_2_O	N/A	900 mL
**Total**	**N/A**	**1,000 mL**

PBS-NaN_3_ storage buffer can be kept in 4°C for up to a year.

**Table:** PBST solution

Reagent	Final concentration	Amount
TritonX-100	0.2%	2 mL
10× PBS	1×	100 mL
dH_2_O	N/A	898 mL
**Total**	**N/A**	**1,000 mL**

PBST solution can be kept in RT for up to a year.

**Table:** Alexa-647 picolyl azide stock solution

Reagent	Final concentration	Amount
Alexa-647 picolyl azide	1.25 mM	1 mg
Fresh DMSO	N/A	744 μL
**Total**	**N/A**	**744 μL**

Aliquot Alexa-647 picolyl azide stock solution. Aliquots can be stored in −20°C for up to a year.

**CRITICAL:** Open a new bottle of DMSO for preparing Alexa-647 picolyl azide solution. Alternatively, aliquot fresh DMSO and store in −20°C for future Alexa-647 picolyl azide solution preparation. Aliquoted DMSO can be stored in −20°C for up to a year.

**Table:** BTTP stock solution

Reagent	Final concentration	Amount
BTTP	20 mM	10.0 mg
DMSO	N/A	1.16 mL
**Total**	**N/A**	**1.16 mL**

Aliquot BTTP stock solution. Aliquots can be stored in −20°C for up to a year.

**Table:** CuSO_4_ stock solution

Reagent	Final concentration	Amount
CuSO_4_	10 mM	5.0 mg
dH_2_O	N/A	3.13 mL
**Total**	**N/A**	**3.13 μL**

Aliquot CuSO_4_ stock solution. Aliquots can be stored in RT for up to a year. Do NOT use PBS to dissolve CuSO_4_.

**Table:** Sodium ascorbate solution

Reagent	Final concentration	Amount
Sodium ascorbate	100 mM	5.0 mg
1× PBS	N/A	252 μL
**Total**	**N/A**	**252 μL**

Sodium ascorbate solution should be prepared fresh each time right before reaction.

**Table:** Click incubation buffer

Reagent	Final concentration	Amount
Alexa-647 picolyl azide (1.25 mM)	5 μM	0.8 μL
BTTP (20 mM)	300 μM	3 μL
CuSO4 (10 mM)	150 μM	3 μL
DMSO	10%	20 μL
PBS	N/A	173.2 μL
**Total**	**N/A**	**∼200 μL**

Click incubation buffer should be prepared fresh for immediate use.

**Table:** Click reaction buffer

Reagent	Final concentration	Amount
Alexa-647 picolyl azide (1.25 mM)	5 μM	0.8 μL
BTTP (20 mM)	300 μM	3 μL
CuSO_4_ (10 mM)	150 μM	3 μL
Sodium ascorbate (100 mM)	2.5 mM	5 μL
DMSO	10%	20 μL
PBS	N/A	168.2 μL
**Total**	**N/A**	**∼200 μL**

Click reaction buffer should be prepared fresh for immediate use.

**Table:** 5 × SSCT

Reagent	Final concentration	Amount
20 × SSC	5×	100 mL
Tween 20 (10%)	0.1%	4 mL
dH_2_O	N/A	296 mL
**Total**	**N/A**	**400 mL**

5 × SSCT buffer can be stored in RT for up to a year.

**Table:** DAPI stock solution

Reagent	Final concentration	Amount
DAPI	10 μM	10 mg
PBS	N/A	2.86 mL
**Total**	**N/A**	**2.86 mL**

DAPI stock solution should be aliquoted and shed from light. Aliquots can be stored in -20 for up to a year.

## Step-by-step method details

### Preparation of mouse brain sample

**Timing: 2 days**

In this step, we will administer PF7845-yne and prepare brain samples. Please note that the transcardial perfusion is the preferred method for preparing conventional brain histology samples. Any conventional protocols for brain perfusion are compatible with CATCH (Gage et al., 2012). For non-brain tissues, please follow standard histology preparation protocols for fixation and tissue dissection.

1. Intraperitoneal (i.p.) administration of 1 mg/kg PF7845-yne in a vehicle of 10% DMSO, 2% Tween-80 in saline with an insulin syringe.

***Note:*** Injection volume is determined as 1% v/w of mouse body weight. For example, for a 20 gram, 6-week-old female C57BL6J mouse, 200 μL drug administration solution (0.1 mg/ml, as above table) should be prepared for injections. 1 mg/kg PF7845-yne was found to fully saturate FAAH. We have tested oral and sub-cutaneous (s.c.) administrations and obtained similar results (Pang et al., 2022).

2. Anesthetize mice with 5% v/v isoflurane mixed with air 1 h after PF7845-yne administration.

***Alternatives:*** Mouse anesthesia can be performed by injecting a mixture of 100 mg/kg ketamine and 16 mg/kg xylazine (Sacma et al., 2022).

3. Perfuse mice with ice cold PBS.
  a. Place one end of pump tubing in ice cold PBS or 4% PFA solution, connect the other end to a 26G needle.
  b. Switch on pump to fill tubing with perfusion solution.
  c. Carefully open chest to expose heart.
  d. Make a small incision at the right atrium. A small amount of dark venous blood should come out.
  e. Insert the needle into the left ventricle, perfuse in ice cold PBS (10 mL/min) to remove blood.
  f. Stop perfusion when the liver is without blood stain and the liquid flowing out is clear. It takes ∼ 2 min (∼ 20 mL PBS) to fully remove blood.

**Figure 2:**
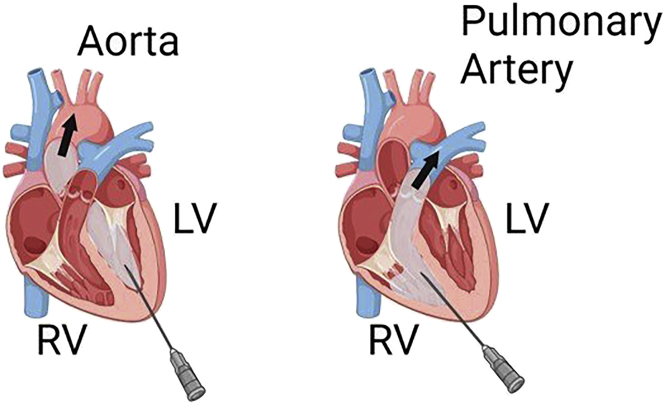
Needle should be placed in the left ventricle (LV) If it punctures into the right ventricle (RV), buffer will enter lung via pulmonary artery.

***Note:*** Effective removal of blood can reduce side reaction in final click labeling (Figure 2, troubleshooting 1). For additional resources on mouse perfusion, please refer to (Gage et al., 2012; Wu et al., 2021).

4. Perfuse mice with ice cold 4% PFA perfusion fixative reagent (10 mL/min) for 2 min till the body gets stiff.

***Note:*** Signs of body twitching, tail flicking and head moving are signs of good PFA perfusion.

5. Decapitate the mouse with tough cut surgical scissors.
6. Dissect out brains, fix samples in 4% PFA perfusion fixative reagent, 4°C, overnight.
  a. Cut skin along the midline. Pull skin to the side to fully expose the skull.
  b. Make two lateral cuts underneath the brainstem.
  c. Cut skull along the midline over the cerebellum.
  d. Insert scissors near the eyes and sever the skull.
  e. Cut skull along the midline over the cortex to fully expose the brain.
  f. Use thumb and index finger to pull skull from the brain. Carefully peel off skull with forceps.
  g. Remove the brain from the skull for PFA fixation.

***Note:*** For additional resources for brain dissection, please refer to (Gage et al., 2012; Wu et al., 2021).

7. Drain PFA. Wash samples with PBS, 10 min, RT.
8. Embed tissue in 2% agarose, keep in 4°C for 1–2 h to get hardened.
9. Slice brains into 100-micron desired sections (coronal or sagittal) with a Leica VT1000S or similar vibratome models.
10. Store brain sections in PBS-NaN_3_ storage buffer, 4°C.

**Pause point:** Uncleared PFA fixed samples can be stored in 4°C for 1–2 months. If storage buffer gets cloudy, samples should be discarded.

### CLARITY tissue clearing

**Timing: 3 days**

In this step, we would perform tissue clearing with CLARITY, a hydrogel-based tissue clearing technique (Chung et al., 2013). CLARITY can remove lipid and render tissue transparent while preserving tissue architecture. Tissue clearing is critical to enable click reaction drug visualization in tissue (Pang et al., 2022). The steps below are adopted from a published CLARITY protocol (Tomer et al., 2014). Please refer to the original protocol for additional details.

11. Prepare A1P4 CLARITY solution on ice. Components for A1P4 CLARITY solution should be pre-chilled in 4°C prior to use.
  a. Weigh out required solid VA-044 in a tube and keep on ice.
  b. Sequentially add water, 10× PBS, 32% PFA, 2% bis-acrylamide, 40% acrylamide solution.
  c. Dissolve VA-044 initiator with ice cold water.
  d. Add dissolved VA-044 and mix solution by shaking.
12. Transfer tissue sections to A1P4 CLARITY solution. Solution should be filled close to top to minimize room for air.

***Note:*** For ∼10 brain sections, use a screw top 5 mL Eppendorf tube. For >20 brain sections, we recommend using a 15 mL centrifuge tube.

13. Incubate sections in A1P4 CLARITY solution, overnight in 4°C with gentle shaking (80 RPM).
14. Connect vacuum desiccator to the vacuum pump and a nitrogen source (i.e., nitrogen tank).
15. Keep tube caps loose on top to facilitate gas exchange. Place tubes in the vacuum desiccator, RT (Figure 3).

**Figure 3:**
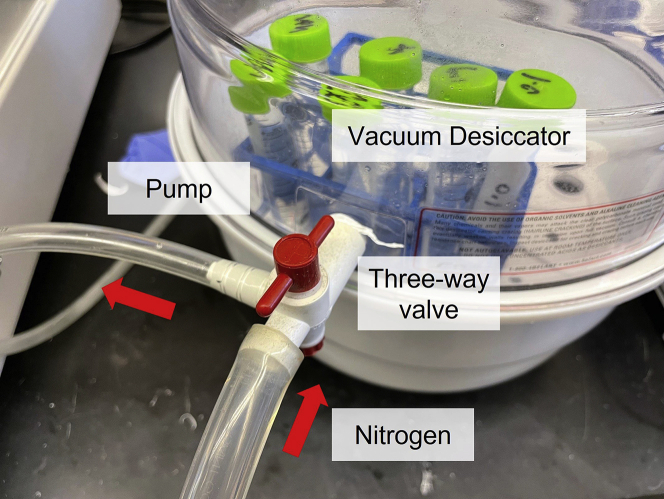
CLARITY degassing set up Samples are placed in a vacuum desiccator. A three-way valve connects the desiccator, pump and nitrogen tank.
16. Switch on pump to remove air, 1 min.
17. Flush in nitrogen from a nitrogen tank or any nitrogen source till the desiccator is filled with nitrogen.
18. Repeat steps 16 and 17 twice to ensure oxygen is fully removed.
19. Close nitrogen tank. Place samples under vacuum for 15 min at RT.
20. Flush in nitrogen. Open the chamber just enough to reach the tubes. With nitrogen flushing, close caps to prevent oxygen entrance.

**CRITICAL:** CLARITY involves a free radical polymerization process and oxygen will inhibit CLARITY polymerization. Ensure oxygen is removed as much as possible.

21. Place samples in a 37°C shaking incubator (80 RPM) to polymerize for 4 h. Tubes should be kept upright to prevent excessive shaking and bubble formation.
22. Drain A1P4 solution. Waste solution should be disposed properly as it contains hazardous materials. Briefly flush tissues with water to remove residue CLARITY A1P4 solution.
23. Clear samples with 8% PBS-SDS, 37°C, 2 days.

***Note:*** We have tested clearing temperature at 37°C–40°C, SDS concentration of 4%–8% and obtained similar labeling efficiency. Higher temperature and prolonged clearing time may cause protein loss and thereby not ideal for thin tissue sections (troubleshooting 2). For additional resources on CLARITY clearing steps, please refer to (Tomer et al., 2014).

24. Wash samples with PBST (PBS with 0.2% TritonX-100, same as follows), 3 times, 10 min each, RT.
25. Wash samples with PBS, 10 min, RT. Samples should look transparent (Figure 4).

**Figure 4:**
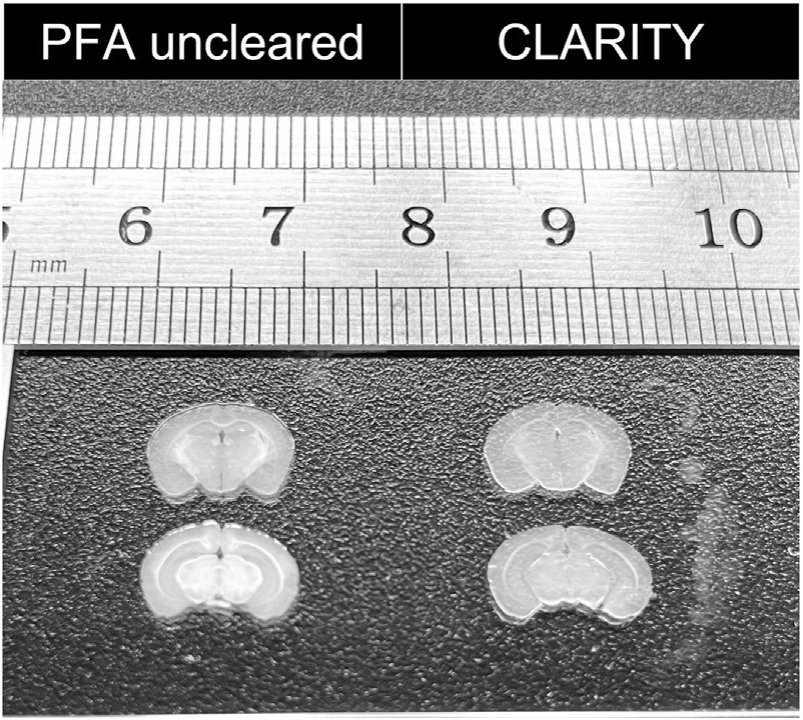
Brain sections after CLARITY clearing
26. Store samples with PBS-NaN_3_ storage buffer, 4°C.

***Alternatives:*** In addition to CLARITY, we have tested other tissue clearing strategies, including but not limited to, SHIELD (Park et al., 2018), iDISCO (Renier et al., 2014), fDISCO (Qi et al., 2019), CUBIC3.0 (Tainaka et al., 2018) and have obtained similar results.

**Pause point:** Cleared CLARITY samples can be stored in 4°C for up to a year without significant difference for labeling efficiency. However, we do recommend refreshing PBS-NaN_3_ storage buffer every 3–4 months to prevent microbial growth.

### Click reaction labeling

**Timing: 2–4 days**

In this step, we would perform click reaction in CLARITY cleared tissue to label drug *in situ* with an Alexa647 fluorescence dye. After click labeling, tissue can undergo secondary staining for molecular target identifications.

27. Prepare click incubation buffer.
  a. Sequentially add PBS, DMSO, 1.25 mM AF647 picolyl azide. Gently pipette to ensure proper mixing.
  b. Pre-mix BTTP and CuSO_4_ stock solution, the solution should turn light blue.
  c. Add Cu-BTTP pre-mixed solution. Gently pipette to ensure proper mixing.
  d. Aliquot click incubation buffer into 2 mL Eppendorf centrifuge tubes (200 μL/tube).

***Note:*** We recommend preparing a master mix with 5% extra volume. For each coronal/sagittal brain section, use 200 μL for incubation. Cu^2+^ concentration can be adjusted between 50–150 μM (Cu to BTTP ratio of 1:2). AF647 picolyl azide has a Cu chelating group and has shown superior reaction kinetics compared to conventional azide tag (Uttamapinant et al., 2012).

28. Transfer cleared tissue to click incubation buffer, 1 section/tube.
29. Place tubes on the tilted tube rack (Figure 1), overnight incubation with gentle shaking (80 RPM), RT. The rack should be shed from light.

**CRITICAL:** Click reaction requires Cu(I) as catalyst. Without reducing agent sodium ascorbate, the incubation step allows Cu^2+^ to diffuse evenly into tissue before reaction. It ensures labeling happens homogeneously across the whole tissue z axis (troubleshooting 3). We do not recommend accommodating multiple tissue sections in the same tube (troubleshooting 4).

30. Prepare click reaction buffer without sodium ascorbate as in step 27.
31. Aliquot into 2 mL Eppendorf centrifuge tubes (195 μL/tube).
32. Transfer tissue sections to click reaction buffer without sodium ascorbate, 1 section/tube.
33. Prepare fresh 100 mM sodium ascorbate solution.
34. Add 5 μL sodium ascorbate solution to each tube (200 μL in total). Gently pipette mix to initiate click reaction (troubleshooting 5).
35. Place tubes on the tilted tube rack (Figure 1). 1 h reaction with gentle shaking (80 RPM), RT. Rack should be shed from light.
36. Quench reaction by adding 0.5 mL, 4 mM EDTA, pH = 8, RT.
37. Immediately transfer samples to PBST. Wash samples with PBST, 3 times, 10 min each, RT to remove click reaction component. Samples should be shed from light.
38. Stain samples with DAPI (1:3,000 dilution in PBS from 10 μM DAPI stock), 15 min, RT.
  ***Optional:*** Samples can now undergo secondary staining for protein and/or mRNA visualization.
  a. FAAH antibody staining.
    i. Incubate click labeled tissue samples in 1: 400 diluted FAAH antibody in PBST, 4°C, overnight.
      ***Note:*** For antibody staining in CLARITY-processed brain sections, blocking is not necessary (Tomer et al., 2014).
    ii. Wash samples with PBST, 3 times, 30 min each, RT.
    iii. Incubate samples in 1:600 diluted Alexa Fluor 488 F(ab’)2 Fragment Donkey anti-Mouse antibody in PBST, RT, overnight.
    iv. Wash samples with PBST, 3 times, 30 min each, RT.
  b. Somatostatin (SST) mRNA hybridization chain reaction (HCR) staining.
    i. Incubate click labeled tissue samples in probe hybridization buffer, 37°C, 30 min.
    ii. Transfer samples to new probe hybridization buffer with 4 nM SST-B1 HCR probe, 37°C, overnight.
    iii. Wash samples with probe washing buffer, 3 × 30 min, 37°C.
    iv. Wash samples with 5 × SSCT, 2 × 30 min, RT.
    v. Incubate samples in HCR amplification buffer, 30 min, RT.
    vi. Prepare hairpin solution in separate tubes. For every 12 μL hairpin, add 4 μL of 20 × SSC. Heat to 95°C for 90 s. Cool to RT in a dark drawer.
    vii. Add hairpin pairs to new HCR amplification buffer to final concentration of 120 nM.
    viii. Incubate samples in hairpin containing amplification buffer, overnight, RT.
    ix. Wash samples with 5 × SSCT, 3 × 30 min, RT.
      ***Note:*** Additional information on HCR protocol can be found on www.molecularinstruments.com.
39. Place samples on a microscope slide. Carefully dry tissue sections with Kimwipe.
40. Immerse sections with RapiClear for refractive index matching.
41. Mount slide with cover glass. Seal cover glass with nail polish and samples are ready for imaging.
42. Confocal microscope imaging of prepared samples. For the listed images here, we imaged our samples with an Olympus FLUOVIEW FV3000 confocal microscope under a 10×, 0.6 NA, water immersion objective (XLUMPlanFI, Olympus), at a z step of 10 microns. Laser setting as 8% power. However, the users should determine their own parameters based on the available equipment and experimental goals.

**Pause point:** CLARITY-based slides can be stored in the dark at RT for at least 1 week. For longer storage, we recommend storing them at the 4°C.

## Expected outcomes

**Figure 5:**
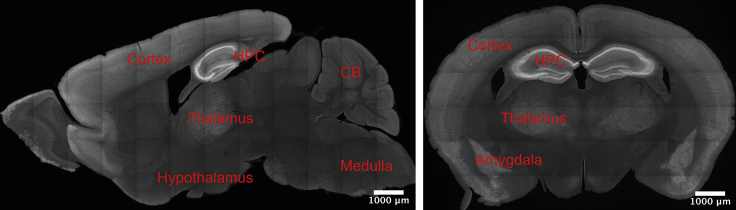
Brain-wide PF7845-yne binding profiles in sagittal (left) view and coronal (right) view PF7845-yne shows binding throughout cortex, thalamus and amygdala. The highest level of drug engagement is found in the hippocampus. HPC: hippocampus; CB: cerebellum. Scale bar, 1,000 μm.

**Figure 6:**
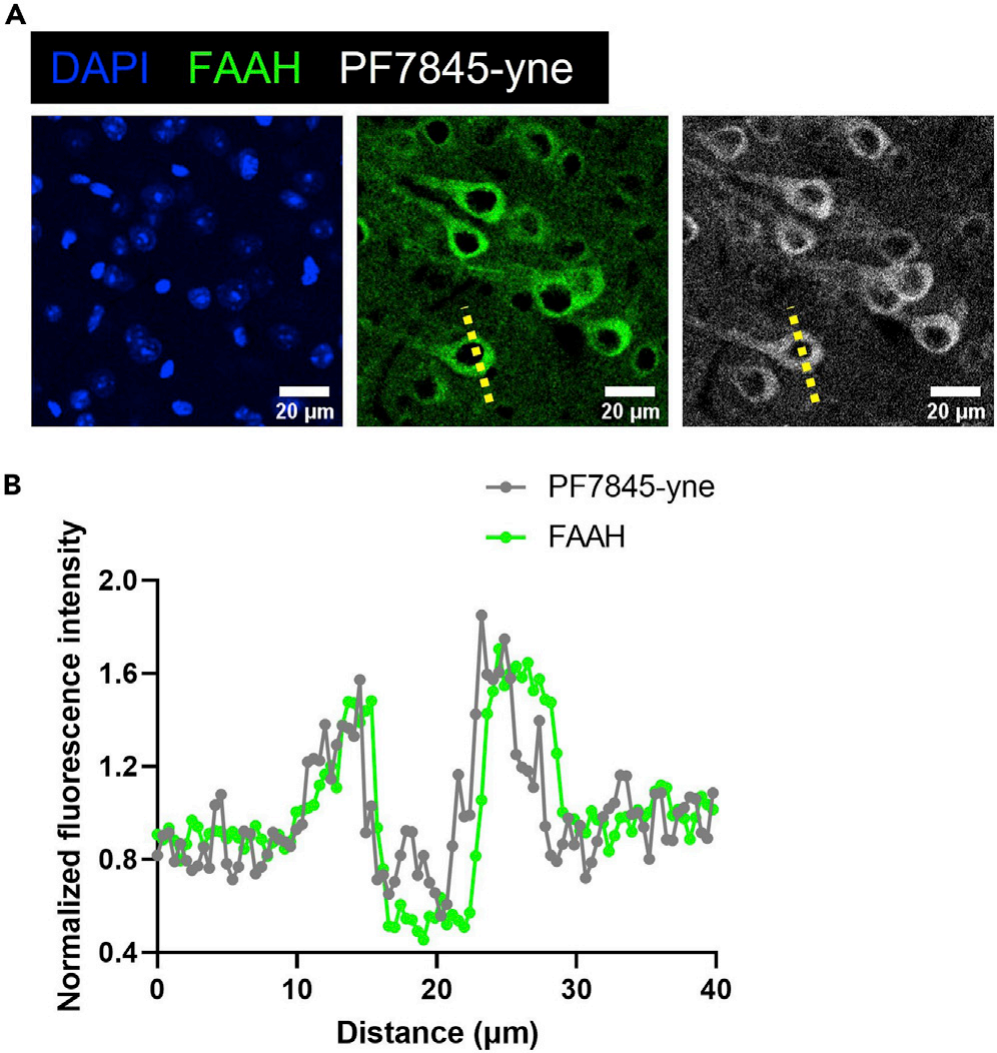
Cellular PF7845-yne binding profile (A) Zoomed in view of PF7845-yne binding taken in secondary somatosensory cortex layer V. FAAH is a membrane protein as shown by immunostaining. PF7845-yne binding is correlated with FAAH expression. Scale bar, 20 μm. (B) Signal profile along the dotted line. Intensity normalized to the mean intensity of all measurements in each channel.

For PF7845-yne, fluorescent drug signal can be observed throughout the cortical region, thalamus, amygdala, with the highest abundance in the hippocampus (Figure 5). As FAAH is a membrane protein (Egertová et al., 2003), drug binding would appear as membrane like structure when examined at sufficient resolution (for example, in Figure 6, at 2.49 micron/pixel in-plane resolution).

## Limitations

CATCH allows for high resolution covalent drug binding mapping in intact tissue. CATCH is highly specific and maps drug binding across different brain regions and cell types. However, as reversible drug-target engagement will be lost during sample preparation, further efforts are still needed to retain reversible drug binding *in situ*. Meanwhile, the protocol is focused on drug imaging in 100-micron brain sections. Further scaling up imaging volume to whole organ, or even whole body, would require optimization in both click reaction and tissue clearing.

## Troubleshooting

### Problem 1

**Figure 7:**
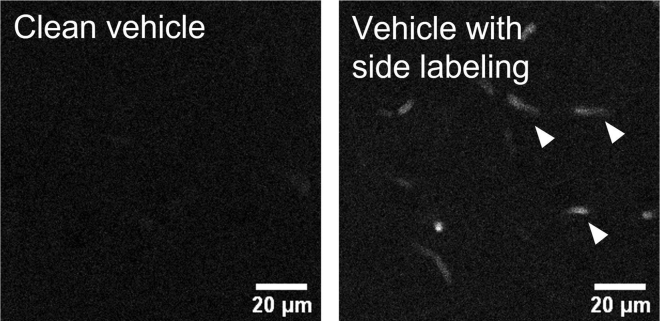
Non-specific click labeling shows blood vessel like structure (arrow sticks) in vehicle controls Images represent primary somatosensory cortex (S1). Scale bar, 20 μm.

Click labeling shows blood vessel like structures in vehicle controls (Figure 7).

### Potential solution

Blood is not fully removed during perfusion. Make sure needle is properly positioned in the left ventricle. Extend PBS perfusion if necessary.

### Problem 2

Tissue deforms during CLARITY clearing.

### Potential solution

CLARITY hydrogel is not well formed to protect tissue structure integrity. Make sure the degassing chamber is properly sealed. Meanwhile, oxygen is inhibiting polymerization, therefore A1P4 solution should fill the tube as much as possible. If vacuum set up is not readily available, consider other tissue clearing techniques including SHIELD, FDISCO, IDISCO and CUBIC3.0, all of which are also compatible with CATCH.

### Problem 3

**Figure 8:**
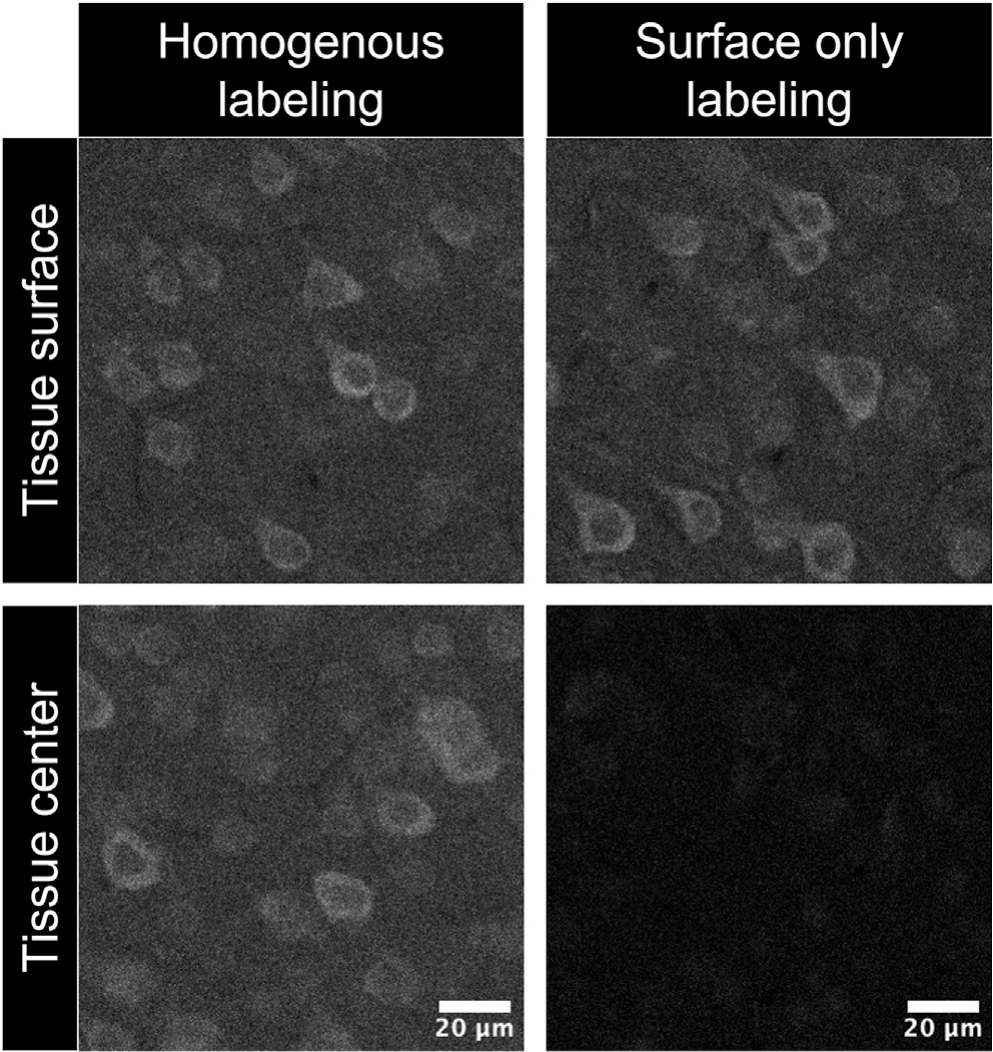
Insufficient click labeling penetration across the Z axis Images represent S1. Scale bar, 20 μm.

Click labeling is not homogeneous across the Z axis, with surface only labeling (Figure 8).

### Potential solution

Refresh CuSO_4_ stock with clean dH_2_O. Perform click incubation at 37°C with agitation. During reaction, increase reducing agent concentration up to 25 mM will further help click reaction penetration.

### Problem 4

**Figure 9:**
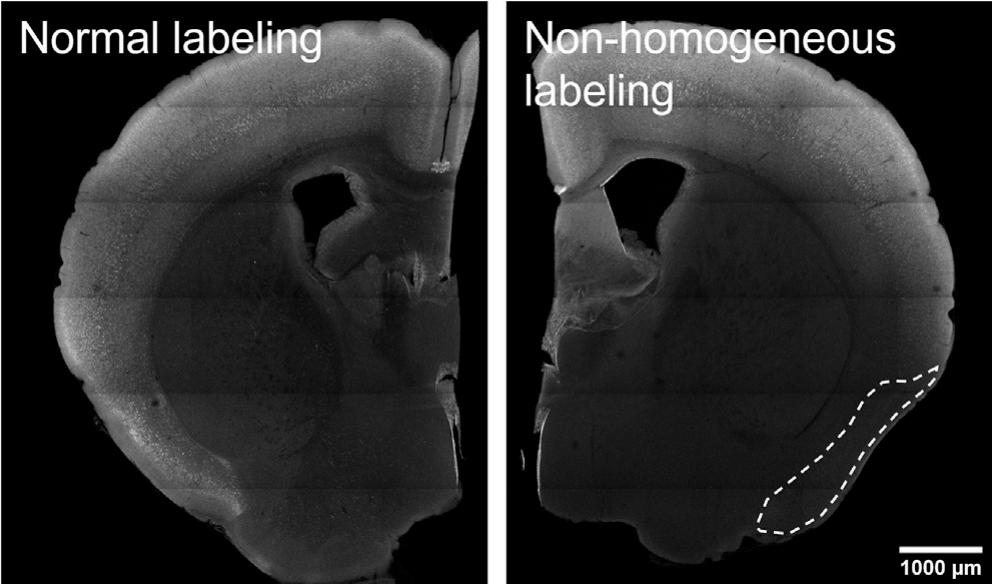
Uneven drug labeling in cortical area Dotted region indicates low labeling area in a failed experiment. Scale bar, 1,000 μm.

Click labeling is not homogeneous on the X-Y plane, with certain parts being dark (Figure 9).

### Potential solution

Tissue is not sufficiently covered by buffer. During both incubation and reaction, the user should make sure all tissue samples are fully submerged in the tube. Ensure sufficient agitation during reaction incubation. Increase incubation temperature to 37°C if necessary.

### Problem 5

Click reaction labeling shows low signal intensity due to low target abundance.

### Potential solution

Optimize microscope laser and acquisition settings. In click incubation and reaction (steps 27–34), increase Alexa-647 picolyl azide concentration up to 20 μM. Increasing CuSO_4_ concentration to 300 μM (Cu to BTTP ratio of 1:2) will further increase labeling intensity.

## Resource availability

### Lead contact

Further information and requests for resources and reagents should be directed to and will be fulfilled by the lead contact, Li Ye (liye@scripps.edu).

### Materials availability

This study did not generate new unique reagents.

## Data Availability

This study did not generate original dataset or code.
